# Do health care professionals’ perceptions help to measure the degree of overcrowding in the emergency department? A pilot study in an Italian University hospital

**DOI:** 10.1186/s12873-019-0259-9

**Published:** 2019-08-27

**Authors:** Andrea Strada, Francesca Bravi, Giorgia Valpiani, Roberto Bentivegna, Tiziano Carradori

**Affiliations:** 1 Emergency-Urgency Medicine Department, S. Anna University Hospital of Ferrara, Ferrara, Italy; 2 Research Innovation Quality and Accreditation Unit, S. Anna University Hospital of Ferrara, Via Aldo Moro 8, (1A3 stanza 3.41.40), 44124 Ferrara, Cona Italy; 3 Medical Direction Department, S. Anna University Hospital of Ferrara, Ferrara, Italy; 4 S. Anna University Hospital of Ferrara, Ferrara, Italy

**Keywords:** Emergency department, Crowding, Healthcare professionals’ perceptions, Perception overcrowding, NEDOCS, Case management, Flow manager

## Abstract

**Background:**

Overcrowding in emergency departments (EDs) is internationally recognized as one of the greatest challenges to healthcare provision. Numerous studies have highlighted the ill-effects of overcrowding, including increased length of stay, mortality and cost per admission. This study measures overcrowding in EDs through health care professionals’ perceptions of it, comparing the results with the NEDOCS score, an objectively validated measurement tool and describing meaningful tools and strategies used to manage ED overcrowding.

**Methods:**

This single-centre prospective, observational, pilot study was conducted from February 19th to March 7th, 2018 at the ED in the University Hospital of Ferrara, Italy to measure the agreement of the NEDOCS, comparing objective scores with healthcare professionals’ perception of overcrowding, using the kappa statistic assessing linear weights according to Cohen’s method. The tools and strategies used to manage ED overcrowding are described.

**Results:**

Seventy-two healthcare professionals (66.1% of 109 eligible subjects) were included in the analyses. The study obtained a total of 262 surveys from 23 ED physicians (31.9%), 31 nurses (43.1%) and 18 nursing assistants (25.0%) and a total of 262 NEDOCS scores. The agreement between the NEDOCS and the subjective scales was poor (k = 0.381, 95% CI 0.313–0.450).

**Conclusions:**

The subjective health care professionals’ perceptions did not provide an adequate real-time measure of the current demands and capacity of the ED. A more objective measure is needed to make quality decisions about health care professional needs and the ability to manage patients to ensure the provision of proper care.

## Background

High demand and need for emergency department (ED) services has been reported worldwide [1, 2]. Indeed, ED overcrowding is increasingly recognized as a global public health problem [3, 4]. Overcrowding in the ED can be the result of several factors, such as entry or “input” problems, internal factors related to “throughput” and exit or “output” bottleneck problems [4]. The delays in transferring patients to hospital wards once triaged and assessed in the ED appear to be an important cause of ED overcrowding, leading to boarding of patients in the ED [5]. Other factors such as the emergence of new healthcare needs, an ageing population, an increasing number of patients with complex cases and the advent of new diagnostic technologies may also contribute to ED overcrowding [6]. For instance, certain subsets of patients have an increased likelihood of boarding in the ED, such as women, elderly patients and patients with severe medical conditions, such as pneumonia or cardiac insufficiency [7]. Regardless of the underlying reason, ED overcrowding can lead to negative patient outcomes, such as possible delays in access to care and diagnosis and increased mortality for patients transferred to hospital wards (for both adult and paediatric patients) and for those discharged from the ED [8, 9]. Placing patients on stretchers in corridors with little privacy, where basic needs such as food and personal hygiene cannot be guaranteed, can also negatively affect a patient’s satisfaction with the care that they receive [10, 11]. Working in such adverse conditions may also compromise public and staff security. Furthermore, there is a high probability of staff “burn-out”, increasing the risk of conflict and further compromising the quality of care [10–14].

Overcrowding has led institutions and health care providers to seek more efficient ways to rapidly administer patient care and make better use of ED waiting times. Several countries, such as the United Kingdom, Canada, Australia and New Zealand, have attempted to reduce overcrowding through dedicated regulatory and managerial interventions. Standards have been defined for maximum duration of stay in the ED (4–6 h), maximum wait time for transfer to a hospital department (2 h), incentives/sanctions for healthcare companies, revision of patients’ hospitalization programmes, centralized management of hospital resources and the development of pre-hospitalization/pre-discharge units [15–18].

In Italy, the structure of the emergency/urgency network is defined by the Ministerial Decree and includes emergency call centres as well as territorial assistance and hospital networks. At the local level, healthcare companies may adopt more flexible guidelines to provide more efficient management of hospital beds in periods of high ED use. In any given year, more than a third (24 million) of the Italian population visits their local ED, and up to 20% of these visits result in hospitalization. ED visits by patients with more complex medical problems have also increased over the years. Those aged 80 and over accounted for 8% of visits in 2005, 10% in 2010 and 12% in 2015. The National Statistics Institute (ISTAT) reported an approximately 60% increase in the aged visiting the ED during this time period, and further increases have been predicted over time, from 1.3 million in 2007 to 4.8 million in 2050. Data for the Emilia-Romagna Region of Northeast Italy are similar to those nationwide. Yearly ED visits have been stable from 2014 to 2015, 2016, and 2017 (1,861,000, 1,857,137, 1,875,560 and 1,891,005, respectively), as have admissions per year (approximately 13%), although there appears to be a steady increase in the time spent by patients in the ED [19].

Several ED crowding estimation tools have been developed and deployed [20–24]. Among these is the National Emergency Department Overcrowding Scale (NEDOCS), which is widely used in the USA [23], as well as in the ED of hospitals in the Emilia Romagna Region [19]. The goal of this study was to measure overcrowding in emergency departments through health care professionals’ perception of it, comparing the results with the NEDOCS score, an objectively validated measurement tool. The goal of this study is to measure overcrowding in emergency departments through health care professionals’ perception of it, comparing the results with the NEDOCS score, an objectively validated measurement tool. The secondary objective is to describe meaningful tools and strategies used to manage ED overcrowding in our University Hospital.

## Methods

This was a single-centre prospective, observational, pilot study. The study was conducted at the Emergency Department in the University Hospital of Ferrara, located in the Emilia-Romagna Region, with a hospital catchment area catering to approximately 340,000 inhabitants. The network has a hub & spoke structure; the hub for STEMI and STROKE is located in the S. Anna University Hospital (Ferrara), and the spokes are community hospitals (Mazzolani Vandini Hospital located in Argenta, SS. Annunziata Hospital located in Cento and Delta Hospital located in Lagosanto, all in the Emilia-Romagna Region). This study covered a sample period of 28 days from February 19th to March 7th, 2018 (study period). The first stage of this study involved data analysis of the NEDOCS scoring tool in determining overcrowding status.

### Assessing overcrowding in the ED

The NEDOCS score is the total of seven variables recorded at a single point in time and entered into a formula to generate the score [25]. These included, as fixed values, 25 ED beds and 528 hospital beds for adults and children, reflecting the installed capacity. The other values used for the score were total patients in the ED, total number of requested hospital admissions from the ED, number of respirators in the ED, longest admission time and waiting room time for the last patient called. The NEDOCS score was calculated every hour at real-time points during the study period and graded as follows: 0–20 not busy, 21–60 busy, 61–100 extremely busy but not overcrowded, 101–140 overcrowded, 141–180 severely overcrowded and 181–200 dangerously overcrowded [23]. The NEDOCS score was compared to the subjective evaluation of ED overcrowding (perceived ED crowding) by the ED staff (physicians, nurses and nursing aides/nursing assistants) Questionnaires were administered by a student/trainee and included the following variables: time at which the questionnaire was answered, role (physician, nurse, nursing assistant), and an index of perception of ED overcrowding rated on a 0–10 cm Visual Analogue Scale (VAS) [26]. The latter reflected the respondent’s level of “feeling rushed” or “pressured” at that particular point in time. The questionnaire was administered twice per shift for three daily shifts (8:00–14:00, 14:00–20:00, 20:00–8:00) during the period from February 19 to March 7, 2018. For comparison purposes with the results of the NEDOCS scale, each individual VAS score was multiplied by 20, as suggested by Weiss et al. [24]. The perception of overcrowding was graded as follows: 0–40 = not busy, 40–80 = busy, 80–120 = extremely busy, not overcrowded, 120–160 = overcrowded, 160–200 = severely overcrowded, and ≥ 200 dangerously overcrowded. The NEDOCS and perceived overcrowding scales were administered during the same time period. The staff participating in the study had over two years of experience working in the ED. Healthcare professionals absent for health reasons (illness, pregnancy, etc.) or on holiday during the study period were also excluded.

### Statistical analysis

The results were expressed as the means and standard deviations (SD) for normally distributed data and as medians and interquartile ranges [1Q 3Q] for skewed data. Categorical variables were summarized using counts and percentages. Quantitative variables were compared among the three healthcare professional groups by an analysis of variance followed by the application of a multiple comparison test or non-parametric Kruskal–Wallis test. Bonferroni correction was used for multiple comparisons. Chi-squared or, when at least one expected frequency in a fourfold table was less than five, the Fisher's Exact test were performed to compare categorical variables (VAS and NEDOCS score graded into six classes, including “not busy”, “busy”, “extremely busy, not overcrowded”, “overcrowded”, “severely overcrowded”, “dangerously overcrowded”) from among groups (physicians, nurses, nursing assistant). Non-parametric Spearman coefficients (r_s_) were calculated to measure the correlation between the two overcrowding measures. The concordance between the overcrowding classification derived from NEDOCS score and healthcare professionals’ crowding perceptions using the six-point VAS scale was evaluated in each ED using unweighted and weighted Cohen’s kappa coefficient and the corresponding 95% confidence interval (95% CI) [27, 28]. The weights assigned were calculated according to Cohen’s method using linear weights (κ_w_) [29]. The κ-coefficient interpretation was performed on the basis of the study by Landis and Koch [30]. The following levels of agreement were considered appropriate for judging the extent of the agreement: κ less than 0.0, poor; 0.0 ≤ κ ≤0.2, slight; 0.21 ≤ κ ≤ 0.4, fair; 0.41 ≤ κ ≤ 0.6, moderate; 0.61 ≤ κ ≤ 0.80, substantial; and κ more than 0.80, almost perfect. Statistical analysis was performed using the statistical package for the social sciences (IBM Corp., IBM SPSS Statistics for Windows, Version 23.0 Armonk, New York USA). We also used the VassarStats website program for Statistical Computation to calculate concordance measurements (http://vassarstats.net/kappa.html). A two-sided value of *p* ≤ 0.05 was considered statistically significant.

## Results

During the study period, 2298 patients attended the ED. Demographic characteristics of these patients and the distribution of emergency colour codes are shown in Table 1. A total of 109 subjects were eligible for the survey: 72 healthcare professionals (66.1%) were included in the analyses, while 37 (33.9%), including 14 physicians, 18 nurses and 5 nursing assistants, did not meet the inclusion criteria. Those who had less than two years’ work experience in the ED (12 physicians, 18 nurses and 3 nursing assistants) or who were absent for health reasons (illness, pregnancy, etc.) or on holiday (2 physicians and 2 nursing assistants) were excluded. The study obtained a total of 262 surveys from 23 ED physicians (31.9%), 31 nurses (43.1%) and 18 nursing assistants (25.0%) and a total of 262 NEDOCS scores. Table 2 reports demographic characteristics, years of work data and NEDOCS and VAS scores in the healthcare professional groups included in the analyses.

**Table 1:** Demographic characteristics of patients in the ED during the study period and distribution of colour codes^a^

		Patients
	*n (%)*	2298
Gender	*female*	1119 (48.7)
	*male*	1179 (51.3)
Age (yrs.)	*mean (±SD)*	61 (22)
	*female*	63 (22)
	*male*	59 (21)
Colour code	*n (%)*	
	*red*	87 (3.8)
	*yellow*	820 (35.7)
	*green*	1306 (56.8)
	*white*	85 (3.7)

^a^The colour code is given to establish the priority of access to treatment based on the seriousness of the case. *Red code*: very critical, danger of death, maximum priority, immediate access to treatment; *yellow code*: fairly critical, high level of risk, potential danger of death; treatment cannot be delayed; *green code*: not very critical, no risk of condition worsening, treatment can be delayed; white code: not critical, not serious and/or not acute

**Table 2:** Demographic, work data and NEDOCS and VAS scores in the three healthcare professional groups involved in the survey

		Physicians N=23 (31.9%)	Nurses N=31 (43.1%)	Nursing assistants N=18 (25.0%)	Total N=72 (100%)
Gender *(female)*†	*n (%)*	11 (47.8)	20 (64.5)	10 (55.6)	41 (56.9)
Age (yrs.) ‡	*mean (±SD)* *min - max*	53.3 (8.4) 36 - 65	43.8 (9.1) 30 - 65	53.9 (4.0) 45 - 59	49.3 (9.1) 30 - 65
Experience working in the ED (yrs.) ‡	*median [1Q 3Q]* *min - max*	15 [9 17] 2 - 26	12 [8 26] 2 - 35	26 [21 32.8] 16 - 35	16 [10 26] 2 - 35
NEDOCS score #	*median* *[1Q 3Q]*	70 [53.5 128.8]	121 [69 162.8]	103 [72.5 148.5]	112.5 [67.8 156]
VAS score §	*median* *[1Q 3Q]*	40 [0 115]	100 [60 160]	100 [60 160]	100 [40 160]

SD: standard deviation; [1Q 3Q] interquartile range; ED: emergency department; † *p* = n.s.; ‡ *p* < 0.001; # *p* = 0.057; § *p* = 0.002

The nurses were younger than the physicians and nursing assistants (*p* < 0.001) and therefore had a shorter median time of work experience than the other groups. The median values of the NEDOCS scores in the three groups showed low statistical significance (*p* = 0.057). Highly significant differences emerged, however, between the median values of healthcare professionals’ perceptions of overcrowding (*p* = 0.002). A positive correlation (r_s_ = 0.614, *p* ≤ 0.001) was found between the objective NEDOCS scale and the subjective scale representing all healthcare professionals. A summary of the extent of concordance between the subjective healthcare professionals’ scale and the NEDOCS score was generated by category and is presented in Table 3. Analysis of the data showed a 28.2% overall concordance with a linear weighted kappa index of 0.381. This indicates poor concordance between the two categorical scores. Nurses had a higher perception of ED overcrowding than physicians and nursing assistants (κ_w_ = 0.403 CI 95% 0.313; 0.493).

**Table 3:** Concordance between the subjective scale (VAS) and objective scale (NEDOCS) by healthcare professionals

	Proportions of concordance		Unweighted Kappa (95% CI)	Kappa with Linear Weighting (95% CI)
	Observed (95% CI)^a^	Change expected		
All healthcare professionals	0.282 (0.230 0.342)	0.174	0.131 (0.065 0.197)	0.381 (0.313 0.450)
Physicians	0.250 (0.114 0.452)	0.162	0.105 (0 0.266)	0.366 (0.213 0.518)
Nurses	0.281 (0.211 0.362)	0.173	0.131 (0.042 0.220)	0.403 (0.313 0.493)
Nursing assistants	0.296 (0.205 0.404)	0.187	0.133 (0.019 0.248)	0.302 (0.168 0.436)

95% CI: 95% confidence intervals. ^a^95% CIs for proportions are calculated according to the Wilson efficient-score method and corrected for continuity

**Fig. 1:**
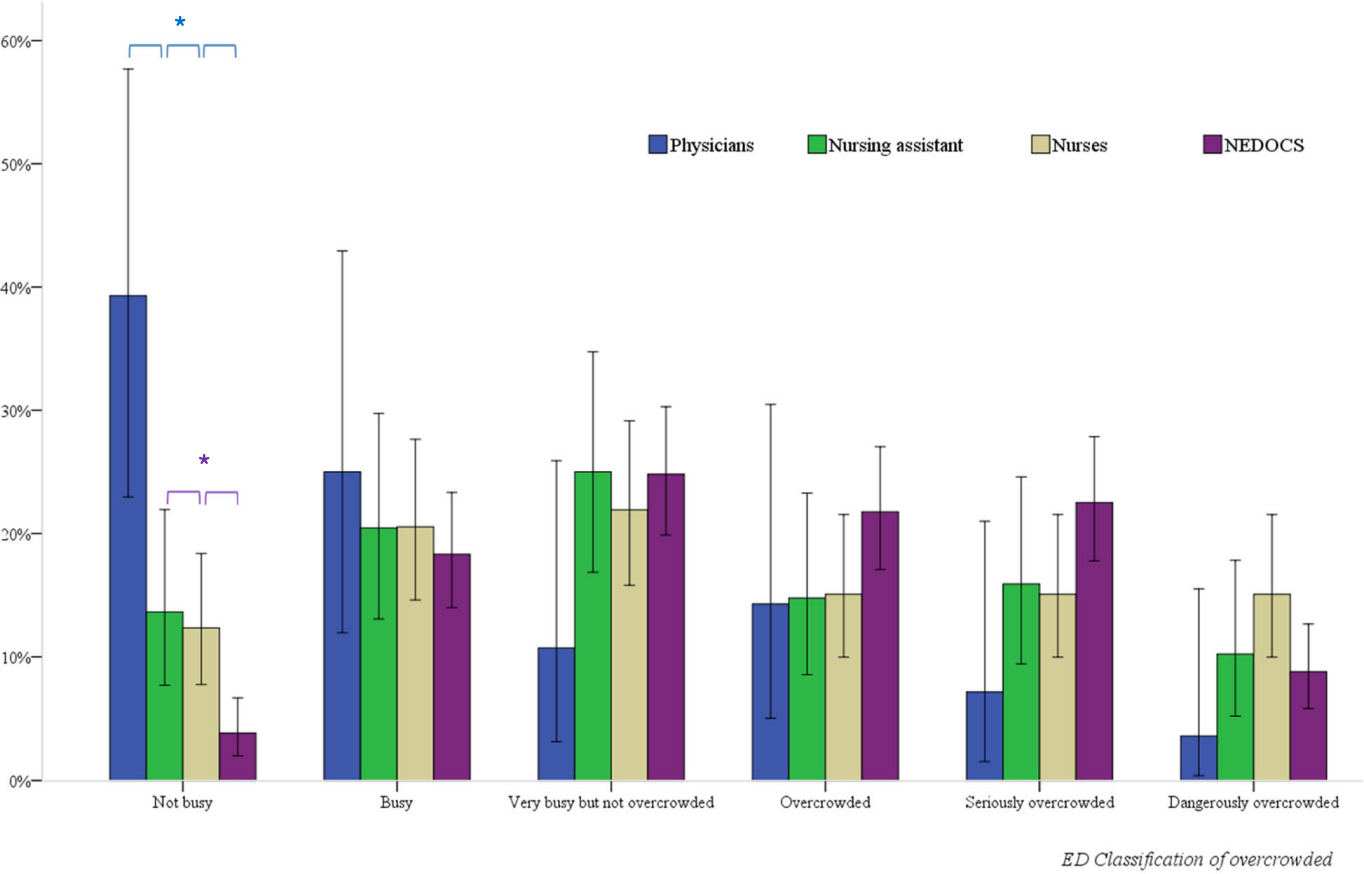
Healthcare professionals’ perceptions of ED overcrowding versus NEDOCS category. Blue bar: Physicians. Green bar: Nursing assistants. Grey bar: Nurses. Purple bar: NEDOCS. Chart with error bars showing proportion values; a blue star indicates statistical significance at level *p* < 0.001 between physicians vs nursing assistant, vs nurses and vs NEDOCS; a purple star indicates statistical significance at level *p* = 0.0001 between nurses vs nursing assistant and vs NEDOCS

Figure 1 compares the distribution of the two 6-point scales, NEDOCS and VAS, and stratifies the objective scale according to categories of healthcare professionals. When the ED was “not busy” or “busy”, the healthcare professionals tended to overestimate the level of overcrowding; in the case of the ED being “not busy”, the difference was statistically significant, particularly for physicians vs nurses and nursing assistants (*p* < 0.001). An examination of the last three classes of overcrowding suggests that the NEDOCS overestimates the subjective values of the healthcare professionals, which, for the “overcrowded” class, have virtually identical values (14% physicians, 15% nursing assistant and 15% nurses). Regarding “seriously overcrowded” and “dangerously overcrowded” classes, physicians had a lower (not statistically significant) perception of overcrowding than nurses (7% physicians vs. 15% nurses for “seriously overcrowded” and 4% physicians vs. 15% nurses for “dangerously overcrowded”). Nurses’ and nursing assistants’ perceptions did not show statistically significant differences in situations of dangerous overcrowding conditions.

## Discussion

Improved tools for early predictors of ED overcrowding could assist EDs and hospital administrators in implementing near-real-time interventions. The NEDOCS score is one tool that is used in Emilia Romagna Region (Italy) and has been found to assess ED overcrowding with relatively high consistency [23]. Overcrowding is considered a perception and can change according to different healthcare professionals’ experience. This study showed little concordance between ED healthcare professionals’ perceived assessments and the NEDOCS score of an overcrowded ED. In general, no common agreement exists between the subjective perception of health care professionals and the objective tool used to measure ED overcrowding [31, 32]. The subjective health care professionals’ perceptions do not provide an adequate real-time measure of the current demands and capacity of the ED. Although health care professionals’ perceptions may provide some useful information, a more objective measurement is needed to make quality decisions about the health care professionals’ needs and the ability to manage patients to ensure the provision of proper care. The values of the subjective healthcare professionals’ perception were not in agreement with the objective NEDOCS scale, particularly when the latter was categorized as “overcrowded” and “seriously overcrowded”. The nurses’ perception of overcrowding was better correlated to the objective scale than the physicians’ perception. This may be because nurses are in closer contact with the needs of the ED and with all the patients, including patients waiting to be treated as well as those waiting to be transferred to a hospital bed. In our study, the physicians felt less “rushed” than the nurses when the EDs were overcrowded, but we did not find a correlation between healthcare professionals’ perceptions and the NEDOCS classification of overcrowding, even when the scales were completed during maximum overcrowding.

Emergency department crowding represents an international crisis that may affect the quality and access to health care. Our solutions to overcrowding include the implementation of a new organizational model to improve hospital “responsiveness” [33].

### Patient flow management in the emergency department

The ED organizational model has been re-examined, and specific monitoring of emergency department functioning has been established, paying particular attention to access, flow and treatment times. Tools and the strategies used to manage ED crowding are described in the Hyperflow management plan (Table 4). The fluidity of the intake pathways for the different priority codes, green, yellow and red will be closely examined. This will be under the supervision of a *Flow Manager* (FM), an experienced nurse responsible for managing patients pre triage, periodically re-examining their clinical conditions in synergy with the clinical–healthcare team and, when necessary, redefining priority codes. Post-triage will also be a concern as the FM would constantly review and redefine priorities in accessing medical care. Furthermore, the FM can improve outflow by acting as the interface between the ED and the various specialized outpatient clinics, the hospital *Bed Managers* (BM) and maintaining a good working relationship with the spoke hospitals of the metropolitan area. BM facilitate correct and swift allocation of patients in the appropriate care settings by checking and controlling activation of the availability of suitable beds. *Case Managers* (CM) are involved in the planned discharge of patients from the ED, assessing the potential risks and difficulties that this process might incur [34].

**Table 4:** Tools and strategies used to manage ED crowding

	input	throughput	output
non-critical situation ^a^	− Agreements between specialists of hospital units in the province for urgent hospitalization in the Hub without the patient passing through the ED − Agreements between specialists of hospital units of the Province for consultations in outpatients’ clinics in the Hub without the patient passing through the ED − Structuring of a Provincial supply of outpatients’ clinics for management of patients with urgent priority for examination within 24 h − Direct hospitalization of a patient attending an outpatients’ clinic via coordination with the bed manager, without the patient passing through the ED	− Implementation of the Flow Manager nursing figure − Structural reorganization of ED with creation of two separate areas: high intensity care and/or high healthcare complexity (patients with red, yellow and green tags) and medium/low intensity care and/or medium/low intensity of healthcare complexity (patients with yellow, green and white tags) − Addition of a team (nurse, doctor) dedicated to high intensity care − Installation of an additional CAT dedicated to the ED function − Creation of an info point in the ED for patients and family members	− Implementation of *bed management* organizational model: structuring of a mixed *operation management* team to guarantee patient flow − Implementation of *case management* organizational model: institution of the *Case Manager* nursing figure in the Medicine Department − Implementation of “Discharge Centre” to assist patients’ return home or accommodation in intermediate care structures (community hospitals, long-term stay structures)
critical situation ^b^	− The same actions as those activated for non-critical situations	The same actions as those activated for non-critical situations	Activation of additional beds in the hospitalization departments of the Hub
seriously critical situation ^c^	Strict control of actions activated in “non-critical situations”	− Activation of an additional daytime team (doctor and nurse) in the ED on working days − Partial freezing of activities programmed in the X-ray department to dedicate more diagnostic time to ED activities − Increase in outsourced services (patient and goods transport)	− Activation of further additional hospital beds in the hospitalization departments of the Hub with respect to the critical situation and request for support from Spoke centres − Freezing of programmed hospitalization intake in the Medicine Department − Referral to private structures accredited with the National Health Service in accordance with supply agreements

^a^NEDOCS score < 140; ^b^140 < NEDOCS score < 180 for 6 consecutive observations; ^c^NEDOCS score > 180 for 3 consecutive observations

The Post-Discharge Follow-Up Centre *(Centrale di Dimissione Continuità Assistenziale* - CDCA) by interacting with primary care and social services, is tasked with activating codified pathways for complex discharges. The CDCA takes factors such as the number of patients awaiting discharge and the total number of occupied hospital into account, helping to safeguard hospital bed resources in the HUB as well as essential services.

The BM, the CM and the CDCA are in constant communication as they share the same objectives of simplifying and facilitating incoming patient flows (*patient – in*), ensuring continuity of care during hospital admission (*patient – stay*) and easing outflow, and maintaining and guaranteeing suitable continuity of care and assistance (*patient – out*). The communicative interfaces of the BM are the ED doctors, the CM, the CDCA and the Medical Department doctors.

### Case management

The CM is a nurse possessing extensive knowledge of the services and organizational structures of the HUB and primary healthcare. The CM ensures appropriate and swift management of hospitalized patients with a view to their ongoing healthcare and facilitated discharge in a suitable primary care setting with proper information. The CM, through updated and precise awareness of the situation of patients held for recovery in the Medical Department, facilitates the use of tools and resources for the planning of discharges. The CM draws on the collaboration of clinical nurses suitably trained to become aware of patients with discharge difficulties and rapidly activates the social/healthcare services of primary care. In such cases, the professionals advise the CM swiftly (24–48 h from hospitalization or as soon as possible) whenever the following occur:

- The hospitalized patient is not self-sufficient, or is likely not to be so following the severe episode in progress, and lives alone or with a fragile family member;
- The patient is a frequent user of hospital structures;
- The patient is accepted by the University Hospital for “social hospitalization”;
- The patient is a guest in a residential structure but is no longer in a condition to return to it;
- The patient is already known to the Home Assistance Services or the Social Services, for which the reason for hospitalization constitutes a worsening of their fragile condition;
- The patient has no fixed address.

The Case Manager refers to the BRASS Index Scale1 for early assessment of a difficult discharge case. The Case Manager interfaces with the professionals in charge of the patient’s case to achieve a planned discharge and simultaneously observe caregivers’ needs for therapeutic education in relation to family members’ conditions and the need to activate aids or devices at home. In the case of necessity, the Case Manager supports activation through the formal structured channels of the CDCA of suitable multidimensional assessments. The Case Manager maintains an ongoing and structured interface with the Bed Manager, informing the latter of the state of discharges from the hospital departments.

## Conclusion

Our results indicate a poor agreement of health care professionals’ perceptions and NEDOCS score that seems to overestimate the subjective values of the healthcare professionals. Our study outcomes indicate that the NEDOCS might not be a suitable tool to determine ED crowding in an academic ED setting, and more objective measurements could be needed to make quality decisions about health care professional needs and the ability to manage patients to ensure the provision of proper care. A larger multicentre study among similar ED environments is required to achieve external validation.

## Data Availability

Not applicable

## Abbreviations

BM: Bed Manager
CDCA: Centrale di Dimissione Continuità Assistenziale
CI: Confidence Interval
CM: Case Manager
ED: Emergency Department
FM: Flow Manager
ISTAT: The National Statistics Institute
NEDOCS: National ED Overcrowding Study
NEDOCS: National Emergency Department Overcrowding Scale
SD: Standard Deviations
VAS: Visual Analogue Scale
